# Editorial: The use of digital technologies in the promotion of children’s health

**DOI:** 10.3389/fped.2023.1304068

**Published:** 2023-10-20

**Authors:** Nan Zeng, Sunyue Ye, Noereem Mena

**Affiliations:** ^1^ Prevention Research Center, Department of Pediatrics, School of Medicine, University of New Mexico Health Sciences Center, Albuquerque, NM, United States; ^2^ Department of Preschool Education, Pinghu Normal College, Jiaxing University, Jiaxing, China; ^3^ Department of Agriculture, Nutrition, and Food Systems, College of Life Sciences and Agriculture, University of New Hampshire, Durham, NH, United States

**Keywords:** digital technologies (DTs), eHealth (electronic health monitoring), MHealth (mobile health), pediatrics, social media

**Editorial on the Research Topic** The use of digital technologies in the promotion of children’s health

Digital technologies, such as smartphones, mobile apps, websites, and text messaging, have become integral parts of children’s everyday lives. They have been confirmed to offer significant health benefits, including improved cognitive creativity, physical well-being, and bio-psycho-social development (1, 2). These technologies provide exciting and innovative methods to enhance knowledge, deliver persuasive messages, modify behaviors, and influence health outcomes. Moreover, they have become more accessible, user-friendly, and widely accepted. In clinical settings, digital technologies have ushered in several noteworthy improvements, enabling real-time data collection beyond traditional clinical contexts and fostering more patient-centered approaches (3). Consequently, digital interventions are increasingly employed to promote healthy behaviors and improve clinical outcomes among pediatric populations. In response to this growing trend, we initiated this special issue to encourage researchers worldwide to exchange and report their recent findings on the application of digital technologies in children and adolescents.

In this special issue, we have gathered a total of five articles from four different countries, presenting a variety of methods and findings that utilize digital technologies to promote and track health behaviors among pediatric populations. Specifically, European scholars have explored the use of digital health technologies, such as eHealth and Telemedicine, in clinical settings. For instance, Poot et al., from the Netherlands employed a participatory approach to develop a novel child-centered eHealth app called “Hospital Hero.” This app aims to reduce preprocedural stress and anxiety among children visiting outpatient clinics by incorporating elements of distraction, gamification, and an animal or hero theme. The Hospital Hero app received positive feedback regarding its use and user experience, making it a valuable tool for healthcare professionals in providing comfort care. Additionally, Martín-Masot et al., examined the impact of the COVID-19 pandemic on the use of digital communication tools, such as Telemedicine apps, among pediatricians in Spain. Their nationwide survey study, involving 306 health professionals, revealed widespread adoption of digital consultations during the pandemic, with pediatricians recognizing the benefits. They concluded that Telemedicine is highly valued by pediatricians and could complement traditional pediatric care in Spain.

Asian scholars, Gaidhane et al., discussed the Rapid-Cycle Evaluation and Learning (REAL) approach to deliver complex and integrated public health interventions in early childhood development programs in rural India. Staff received training in using a measurement matrix developed by the research team. By analyzing and triangulating data in various forms, the authors observed a 50% reduction in data collection errors, along with improvements in narrative quality, making it a valuable tool for enhancing the confidence and engagement of frontline service providers in resource-limited settings. Furthermore, Liu et al., from China examined the association between problematic smartphone use (PSU) and blood pressure (BP) in children and adolescents. Their experiment included 2,573 participants from 14 middle/high schools in Shanghai. The results showed a positive relationship between PSU and high BP, with variations based on student grade and dimensions of PSU, such as social network usage, entertainment, and compulsive behavior. Finally, Pi et al., from China conducted a systematic review and meta-analysis of randomized controlled trials (RCTs) to assess the impact of smartphone apps on glycemic control in young patients aged 0–24 years with type 1 diabetes (T1D). Their analysis of nine articles involving 748 participants did not reveal a significant reduction in HbA1c with smartphone app use in combination with usual care. However, auxiliary-style apps with insulin or carbohydrate calculators showed promise in reducing HbA1c.

In summary, the articles in this special issue have highlighted (1) the potential of eHealth and Telemedicine in clinical settings, (2) the effectiveness of real-time data collection and analysis systems for improving the quality and coverage of complex public health interventions, (3) the impact of problematic smartphone use on children’s blood pressure, and (4) the effect of smartphone applications on glycemic control in young patients. These findings underscore the growing application of digital technologies in clinical and non-clinical settings, offering cost-effective solutions. Digital technologies have been widely used for health promotion and disease prevention in general populations (4), they also hold promise for enhancing clinical outcomes (5). Their application in pediatric populations, however, remains relatively unexplored, despite the growing use of mobile apps, tablets, wearable devices, and educational video games to promote children’s health behaviors (6). Hence, we encourage further research to delve into the best use of digital technologies in children, especially in infants, toddlers and preschoolers, as research in this field remains limited. All these endeavors require a comprehensive understanding of the intricate aspects of the technologies that shape our digital society.
